# On the Cause of Death under Chloroform

**Published:** 1882-02

**Authors:** G. P. Hachenberg

**Affiliations:** Austin, Texas


### ON THE CAUSE OF DEATH UNDER CHLOROFORM.

BY G. P. HACHENBERG, AUSTIN, TEXAS.

The subject of this paper was forcibly brought to my mind in connection with my researches on the malignancy of epidemic diseases, as cholera, yellow fever, etc. The similarity of action of anæsthetics and some of the chemical and epidemic poisons on the system led me to think that there was a common cause in their disastrous results. Take into consideration almost any active chemical poison, as arsenic, morphia, etc. A few grains may be sufficient to destroy life. But let a person take small medici nal doses of the same daily, and gradually increase them, he may finally be able to take ten times the quantity with impunity that would have proved fatal at the first dose. Here is a physiological principle involved that should solve the difficulty of our question. Let the nervous centers be *suddenly* overwhelmed with any poison, bad results will follow. An epidemic poison that is gradually admitted into the system will invariably produce a mild type of disease, where on the other hand, if the absorbents take it up suddenly, it is sure to endanger life.

The cause of death under chloroform is not usually owing to any organic defect in the system, as it is usually supposed, but to the condition of the absorbents. If the functions of these are in a measure suspended, chloroform, if administered in the ordinary way, would hardly ever prove fatal. But let them be in a ravenous condition, with a corresponding clogged elimination, when chloroform is administered like any other poison, it is apt to prove fatal. As to the mode of dying under the influence of chloroform, that is entirely a secondary matter, and comes within the range of some constitutional defect that may exist at the time. This explains the want of pathological harmony of those fatal cases. I do not agree with Richardson, that "no human skill in applying chloroform can divest it of its danger." I believe there are two ways of accomplishing this:

1st. By administering chloroform daily, sparingly every day, for several days before its final administration.

2d. By counteracting the activity of the absorbents.

There are circumstances when the latter is enforced by the process of nature alone, as in certain forms of injuries and in all cases of parturition. Consequently cases under this peculiar condition are not likely to prove fatal. In my army experience, I have noticed that a healthy, vigorous subject in a fast, with great fatigue, cannot take chloroform with safety, and for obvious reasons.

The question might be asked, if chloroform is not usually received into the system by the absorbents, how is it received? Precisely as the oxygen of the air is admitted—by a limited pulmonic endosmosis, virtually a mechanical process. Instantaneously as the anæsthesia makes its impress on the brain, insensibility ensues. Should this process of endosmosis into the pulmonic capillary arteries be associated with an active absorption by the veins, the venous portion of the heart would become paralyzed, thus causing death.

There is another idea connected with this matter that deserves serious attention. A self-generating poison in the system, as in smallpox, scarlatina, etc., is governed by the same law as any extraneous poison that may be received into the body.

